# Preliminary needs assessment of mobile technology use for healthcare among homeless veterans

**DOI:** 10.7717/peerj.1096

**Published:** 2015-07-30

**Authors:** D. Keith McInnes, Gemmae M. Fix, Jeffrey L. Solomon, Beth Ann Petrakis, Leon Sawh, David A. Smelson

**Affiliations:** 1 Department of Veterans Affairs, Edith Nourse Rogers VA Hospital, Bedford, MA, USA; 2 Department of Health Policy and Management, Boston University School of Public Health, Boston, MA, USA; 3 VA National Center on Homelessness among Veterans, Philadelphia, PA and Bedford, MA, USA; 4 Department of Psychiatry, University of Massachusetts Medical School, Worcester, MA, USA; 5 School of Criminology and Justice Studies, University of Massachusetts, Lowell, MA, USA

**Keywords:** Health informatics, Veterans, Homeless persons, Mobile phones, Access to care, Health information technology, Vulnerable populations, Outreach, Engagement in care

## Abstract

**Background.** Homeless veterans have complex healthcare needs, but experience many barriers to treatment engagement. While information technologies (IT), especially mobile phones, are used to engage patients in care, little is known about homeless veterans’ IT use. This study examines homeless veterans’ access to and use of IT, attitudes toward health-related IT use, and barriers to IT in the context of homelessness.

**Methods.** Qualitative interviews were conducted with 30 homeless veterans in different housing programs in Boston, MA, ranging from emergency shelters to supportive transitional housing that allow stays of up to 2 years. Interviews were conducted in person, audio recorded and then transcribed. Three researchers coded transcripts. Inductive thematic analysis was used.

**Results.** Most participants (90%) had a mobile phone and were receptive to IT use for health-related communications. A common difficulty communicating with providers was the lack of a stable mailing address. Some participants were using mobile phones to stay in touch with providers. Participants felt mobile-phone calls or text messages could be used to remind patients of appointments, prescription refills, medication taking, and returning for laboratory results. Mobile phone text messaging was seen as convenient, and helped participants stay organized because necessary information was saved in text messages. Some reported concerns about the costs associated with mobile phone use (calls and texting), the potential to be annoyed by too many text messages, and not knowing how to use text messaging.

**Conclusion.** Homeless veterans use IT and welcome its use for health-related purposes. Technology-assisted outreach among this population may lead to improved engagement in care.

## Introduction

The health of homeless veterans is among the worst of any vulnerable group, which is concerning given that there are approximately 49,900 homeless veterans on the street (US Department of Housing and Urban Development, 2014) and homeless veterans comprise 11% of the US homeless population (Perl, 2011). Homeless veterans have high rates of chronic conditions. A study of urban homeless veterans receiving care in a Department of Veterans Affairs (VA) medical center found 53% suffering from degenerative joint disease or arthritis, 45% with hypertension, 35% with hyperlipidemia, and 28% with hepatitis (O’Toole et al., 2010). The study also reported that mental health related conditions were highly prevalent, with 71% suffering from alcohol use disorder, 67% with depressive disorders, 43% cocaine use disorder, 37% anxiety disorders, 17% opioid use disorder, and 11% bipolar disorder.

Information technologies (IT) are increasingly being used to improve access to health care, make utilization of services more efficient, and improve health outcomes (Free et al., 2013; Cole-Lewis & Kershaw, 2010). IT such as mobile phone tools including texting and apps contribute to improved medication adherence (Lester et al., 2010), increased clinic attendance rates (Guy et al., 2012), increased vaccination rates (Stockwell et al., 2012), and behavior changes such as reduction in smoking in non-homeless populations (Free et al., 2011). There is early stage research into the use of mobile phone apps to promote substance use harm reduction and abstinence (Cohn et al., 2011). Survey data from 2010 indicate 71% of veterans use the internet and 20%–25% use the VA’s electronic personal health record system (PHR) (Tsai & Rosenheck, 2012). While some of this research examines use of information technologies with people with low incomes in low resource settings, such as in developing countries, there have been few interventions aimed at assisting homeless persons in their use of technologies for health-related purposes. Data from primarily non-veteran populations indicate that about half or more of homeless persons have mobile phones (Eyrich-Garg, 2010; Stennett et al., 2012; McInnes, Li & Hogan, 2013). Therefore, we sought to understand homeless veteran’s access to and use of information technologies, and whether using these technologies to communicate with health care providers would be acceptable to them. While we sought information on a wide range of technologies (e.g., computer, mobile phone, internet), special attention was given to the topic of mobile phones, for two reasons: first, the growing literature indicating that persons who are homeless have access to that technology more than others (McInnes, Li & Hogan, 2013; Eyrich-Garg, 2010; Eyrich-Garg, 2011), and second, the VA’s development of a patient-provider text messaging system for chronic disease management (McInnes et al., 2014). This system could be a promising means of improving patient-provider communication and addressing chronic health conditions in homeless veterans. Veterans are an important group to study because of the multiple opportunities to engage them in their health care through existing and developing information technologies, such as the My HealtheVet personal health record system used by 1.8 million veterans (Nazi et al., 2013), and the coming national patient-provider text messaging system (McInnes et al., 2014). Though the study sample is veterans, there are reasons to believe that lessons learned will be transferable to non-veteran homeless populations. Both populations share many of the same health care needs, barriers to health care services, and challenges accessing information technologies (Tsai & Rosenheck, 2012; O’Toole et al., 2003; Tsai, Mares & Rosenheck, 2012). Our research questions were: (1) How accessible are information technologies (e.g., computers, internet, and mobile phones) to homeless Veterans?; and, (2) What level of interest do homeless veterans have in using information technologies to communicate with healthcare providers?

## Methods

The current qualitative study was one component of a larger survey project that examined rates of technology ownership among 106 homeless veterans living in an urban area of the Northeast US (McInnes et al., in press). In the current study, we used a qualitative approach that was based on grounded theory (Glaser, 2000). This involved inductive analysis and coding that used the constant comparative method (Glaser, 1965), with an emphasis on allowing the emergence of themes from the data. In-depth interviews were conducted with 30 homeless veterans to explore access to, and perceptions of, information technologies, as well as attitudes toward using information technologies for health related purposes.

### Participants

We sought to represent a variety of homeless veterans by recruiting from different geographic locations and housing programs around the greater Boston metropolitan area. Thus, our sample represents an urban population. Our target was to interview 20 subjects, expecting that would achieve theoretical saturation (Guest, Bunce & Johnson, 2006). Additionally we wanted diversity of subjects that might be enhanced by recruiting subjects from four different types of housing programs. We sought to interview a minimum of five per organization, but were inclined to have larger numbers, if feasible, because this would add to our knowledge of the different types of housing programs and potential roles they played in facilitating or impeding the residents’ use of information technologies. At each of the organizations, we exceeded our targets because staff facilitated the recruitment and interview process; for example by making 2 interview rooms available for the researchers. The 30 veterans recruited came from five locations representing four different types of housing programs—domiciliary, transitional housing, grant-per-diem (GPD), and emergency shelter (we recruited from two shelters that were in adjoining towns, but run by the same organization). Two housing programs (domiciliary and transitional housing) were run by the US Department of Veterans Affairs (VA) and two by a single local non-profit organization (GPD and the two emergency shelters). The VA Domiciliary program has a maximum 100 day stay with a focus on veterans with substance use disorders, while the VA transitional housing program allows residence for up to two years. GPD refers to housing owned by a non-profit, with maintenance and program costs partially subsidized by federal funding. Residents in the GPD program described in this study can stay for up to two years and they receive supportive services including case management and vocational training. Sampling from these different programs was intended to provide representation of a variety of health care and other needs. Emergency shelters, for example, tend to serve persons with fewer connections to services and more unstable lives (Petrovich, Pollio & North, 2014), while GPD programs typically require more evidence that a person has made substantial progress in recovery from substance use and has more supportive services that help maintain stability (Gabrielian et al., 2014; VA Homeless Providers Grant and Per Diem Program, 2013).

Researchers met with staff at the four programs to describe the study objectives and to enlist staff assistance in notifying residents about the study. In addition, at the domiciliary and the transitional housing facilities, investigators described the study directly to residents at the weekly resident meetings and posted flyers about the research study. There was no attempt to oversample women or racial/ethnic minorities. Interviews were conducted between January and September 2012 at the program facilities. Prior to the interviews, a qualitative team investigator (GF) conducted training for interviewers (BAP, KO, DKM) on semi-structured interviewing techniques and taking field notes.

Written informed consent was obtained from all participants prior to the interviews. Participants received $25 for study participation. The study conforms to the Declaration of Helsinki ethical principles and was approved by the Institutional Review Board of the Edith Nourse Rogers Memorial Veterans Hospital, Bedford, Massachusetts (Approval #0008).

### Data collection

We conducted semi-structured qualitative interviews. In addition to collecting demographic and health information, interview topics covered two broad areas, first, access to IT, including ownership of a mobile phone, use of mobile phones, uses of other information technologies (e.g., computer, Internet), and information technology use related to health care; and secondly, perceptions of potential health-related interventions based on mobile phone calls or texting, such as reminders (e.g., for appointments, medication-taking and refills, availability of lab results). Interviewers recorded descriptive field notes immediately following interviews, using a template designed to capture detailed information about the interview that would be available for discussion by the team at weekly meetings (Bernard, 2002). The template had the following categories: overview (e.g., general impressions, interview rapport), description of participant (e.g., affect, how dressed), highlights (memorable parts of the interview), key thoughts about technology and interventions (e.g., how might a health information technology intervention work for someone like this), life issues, and barriers and facilitators (both to information technologies and to healthcare). Completing the notes was done in conjunction with listening to the audio. The field notes included quotes or paraphrases (and minute marker so that it would be easy to find the quote on the audio). These detailed field notes both helped to capture information about the interview that is not picked up well by audio (e.g., perceptions of mood or personality of the respondent) and provided the first step in the analysis process, without having to wait for all audio recordings to be transcribed. In addition, verbatim interview transcripts were created and used by the research team to extract participant quotes to augment what was in the field notes.

### Analysis

An analysis team (BAP, JS, KM) led by a qualitative research expert (JS) conducted an inductive thematic analysis to identify broad themes and subthemes from the field notes (Patton, 2002). Investigators used an iterative process to develop a preliminary list of themes and to develop inter-rater reliability. Each member conducted a close reading of seven field notes and coded them for preliminary themes. Through in-person meetings, the team developed consensus on the list of themes. Then, using a set of 5 field notes, team members did coding, compared coding results, discussed differences and came to consensus about the codes, as a means of achieving inter-rater reliability. Meetings were used to discuss those themes, determine whether they indicated the emergence of additional themes, and make a revised list of themes. Through subsequent meetings some themes were collapsed and new themes were added. The remaining 18 field notes were divided among the analysis team for coding, with investigators meeting regularly to discuss the coding process and any potential new themes. Throughout these discussions, investigators maintained a strong degree of consensus regarding the themes. Themes and corresponding examples from field notes were documented and tracked in a shared folder accessible to the analysis team.

Once all field notes had been coded in the shared coding document, each member of the analysis team developed a written summary of the most salient themes. Salience was defined both by frequency of codes supporting that theme and/or the degree that it addressed study goals. We met to discuss the written summaries and come to consensus on the most important findings. In a final phase, after developing preliminary interpretations, we searched the data for alternative interpretations and rival conclusions.

## Results

### Description of participants

The 30 participants ranged in age from 33 to 65 years. Most (87%) were male and white (77%). Other race/ethnicities were black (17%) and Native American (7%). Over half (60%) had some college education (but not 4-year degrees), 37% had completed high school or equivalent, and 3% had not completed high school. Almost all (90%) had a mobile phone at the time of the interview, 70% used the Internet, and 72% had an email address. See Table 1. Of the cell phones, 30% were smartphones. When asked about current health, commonly mentioned conditions were depression, PTSD, substance and alcohol use disorders, anxiety, and hepatitis C.

**Table 1:** Characteristics of study participants.

	VA domiciliary (*n* = 9)		VA transitional housing (*n* = 6)		Emergency shelters (*n* = 9)		Grant per diem (*n* = 6)		Total (*n* = 30)	
	%	(*n*)	%	(*n*)	%	(*n*)	%	(*n*)	%	(*n*)
**Male**	78	(7)	83	(5)	100	(9)	83	(5)	87	(26)
**Ethnicity**										
Hispanic	0	(0)	0	(0)	11	(1)	17	(1)	0	(0)
Native Am	11	(1)	0	(0)	0	(0)	0	(0)	7	(2)
Black	0	(0)	50	(3)	11	(1)	17	(1)	17	(5)
White	89	(9)	50	(3)	78	(7)	67	(4)	77	(23)
**Education**				()						
Some HS	0	(0)	17	(1)	0	(0)	0	(0)	3	(1)
HS/GED	22	(2)	50	(3)	33	(3)	50	(3)	37	(11)
Some college	78	(7)	33	(2)	67	(6)	50	(3)	60	(18)
**Have cell phone**	78	(7)	100	(6)	89	(8)	100	(6)	90	(27)
**Go online**	78	(7)	67	(4)	56	(5)	83	(5)	70	(21)
**Have email**	67	(6)	67	(4)	56	(5)	100	(6)	70	(21)
**Age, Mean** (SD)	50.7	(10.16)	54.8	(5.12)	58.0	(4.64)	50.0	(10.49)	53.6	(8.34)

**Notes.**

VA Department of Veterans Affairs SD standard deviation HS high school GED high school equivalency exam

### Overview of findings

We have organized our findings into four main areas: (1) Barriers to communication with health care providers; (2) Access and barriers to, and current use of, information technologies, in general; (3) Current uses of information technologies for health-related purposes; and, (4) Attitudes toward information technology use for new types of health-related communications. Related to the fourth theme (attitudes toward information technology), our interview questions covered specific uses of IT for appointment reminders, medication refill reminders, medication adherence support, and laboratory result notification—all of which have been tried in the general population, but not with homeless persons. Findings from each of the four main areas are described below.

### Barriers to communication with health care providers

Becoming homeless creates substantial disruption in a person’s life that can be long lasting. Communication with health care providers and systems becomes complicated. Some reported that they no longer had a reliable mailing address, or the health system had an old address on file and letters were going to that address. Thus, they sometimes missed important letters containing medical appointment reminders or changes, or laboratory results. Similarly, most no longer had a landline phone as a result of their homelessness, causing them to miss telephone appointment reminders from their healthcare providers. Cell phones replace landlines, but participants staying in shelters reported that guests must hand in their mobile phones to staff in the late afternoon or evening for safekeeping overnight.

### Access and barriers to, and current use of, information technologies in general

Information technologies (e.g., computers, mobile phones, tablets) were used by almost all study participants. They used these devices for many of the same reasons that non-homeless persons do, such as staying in touch with family and friends, taking care of personal business (e.g., making appointments, checking bank accounts, connecting with health care providers), entertainment, and gathering information, including, to some extent, health-related information.

#### Mobile phones

Ninety percent of participants had mobile phones. Participants paid for the devices and service in a number of ways. Some got their phones through government programs that provide a free device and 250 free minutes per month, with some respondents noting they had 2 such free phones in order to boost the number of monthly available minutes. Others had inexpensive devices and bought pre-paid cards (e.g., $10 or $20) for phone and/or texting services and reloaded cards as needed. Others were on contracts. Typically this was through a relative, friend, spouse, or ex-spouse. Mobile phones were used for things like keeping in touch with family and friends, tracking appointments on the calendar feature, and searching for work. A number reported they used texting, while others did not, but indicated they would like to learn how.

#### Computers and internet

Use of computers, the internet, and email were common. A few had laptops, while others reported using computers at libraries and other locations that provided free computer and internet access. Common uses were for reading the news, searching for jobs, and entertainment, such as listening to music and playing games. Some used the Internet to find places to stay. A few reported not knowing how to use the internet and similarly not having an email account. However, there was interest among these respondents in learning how to use the internet.

### Current uses of information technologies for health-related purposes

#### General uses

Many participants were using computers, Internet, and mobile phones for health-related purposes. Table 2 summarizes our findings related to perceptions and use of IT for health-related purposes. Some seemed to regularly use their mobile phones to keep in touch with primary care and other health care providers, including psychiatrists, social workers, and case managers.

**Table 2:** Perceptions and uses of IT for health-related purposes, summary of findings.

Topic	Perceptions and uses (*exemplar quotes in italics*)
Current health-related uses of technology	• Receive cell-phone reminder calls for appointments
	• Use of mobile phone to stay in touch with health professionals
	• Some reported dislike for automated appointment reminder calls which can be confusing and use up valuable cell phone minutes.
	*“(When you receive an automated call) …you can’t shut it off and you’re stuck with it and it eats up all your (phone minutes) and you don’t know who it is.”*
Perceived benefits	• Convenience: information is retrievable, there is less need to write appointment information down, and the asynchronous communication is less intrusive.
	*Well you have something solid in front of you. You don’t have to write it down. You can save it and it’s there. I mean you have all your information right there.*
	• Organization: reduces worry about losing slips of paper with appointment information, and forgetting to go to appointments
Possible barriers	• Cost: calls and texts cost the recipient money if they do not have an unlimited mobile phone plan
	*“(I wouldn’t want cell phone reminders)…not with what it costs me.”*
	• Annoyance: too many text messages become annoying instead of helpful
	• Lack of skills: some participants lacked text messaging skills

*“I’d be lost (without my cell phone) …I keep in close communication with my social worker, for my doctors, and everything. They always check on me …”*

A few used mobile phone text messaging to contact health care providers, including to find out about upcoming appointments. Many respondents used the internet to look up information about medical conditions, medications, side effects, and medication interactions. Some used email to check upcoming appointments with health care providers. Few respondents reported ever having used an electronic personal health record (PHR), which, in the VA, is called My HealtheVet. One reported using the My HealtheVet PHR to order medication refills and to check laboratory results, and several others knew about the My HealtheVet PHR but had not used it. Quite a few had never heard of it.

#### Appointment reminders, medication refill reminders, medication adherence, and laboratory results

Respondents were asked about whether they currently received reminders via mobile phone or Internet, about appointments, medication refills or medication taking, or if they received notification that laboratory results were ready. Some respondents currently received phone call reminders and liked them. There was variation in what kind of phone calls the respondents liked. Several liked live (e.g., speaking directly to receptionist or nurse) reminders because they were seen as more personal and allowed patients to ask questions. Several respondents received automated calls, or “robo-calls,” but they generally were not satisfied with them. They reported they could be confusing, for example it would not be clear to which clinic or doctor the appointment was for, and there was often no phone number given to call back. None were currently receiving regular text message reminders for health related purposes.

No respondents reported receiving systematic reminders to refill a prescription or to take a dose of a current medication. Laboratory results, respondents reported, were generally given to them in-person when they came for health care visits. Respondents did not report receiving mobile phone calls or text messages to let them know their laboratory results were ready.

### Attitudes toward information technology use for new types of health-related communications

We explored with participants a variety of ways that healthcare providers might use mobile phones to communicate with patients. We asked respondents about their interest in receiving (1) appointment reminders, (2) medication-related reminders, (3) notifications that laboratory results were ready, and (4) “checking-in” type outreach (either daily phone calls or texts to ask “how are you,” or phone calls or text messages to patients who had not been seen in the clinic in a long time to encourage them to come for a visit). There was considerable support for healthcare providers using mobile phone technology to communicate with veterans for these kinds of purposes.

#### Appointment reminders

There was strong interest in receiving appointment reminders from healthcare providers (Table 3). Many respondents cited poor memory as one of the reasons that reminders were appealing.

**Table 3:** Attitudes toward specific health IT communication tools.

Topic	Attitudes and perceptions (*exemplar quotes in italics*)
Appointment reminders	Many were supportive of text messages used for appointments *Well you have something solid in front of you. You don’t have to write it down. You can save it and it’s there. I mean you have all your information right there.*
Medication-related reminders	Support for parsimonious use of medication related-reminders *If they (send text messages) more than once I would get annoyed. You know, …if they do it like four, five times …I’ll probably throw the phone up against the wall!*
Notifications related to laboratory work needed and results	Support for text message reminders to get laboratory tests done *(Text messages to remind you to have lab work done) would be very helpful because they usually schedule (lab work) like months in advance. And if you don’t write it down on the calendar and you try to remember something like that, it’s impossible. So that would be a very good idea.*
“checking-in” type outreach	Proactive messages to patients not seen in clinic for a long time *That would be a huge help and then if you say, “no,” (I’m not doing well) well okay then they transfer you and then either you’re texting or phoning with somebody to try to help get you immediate help. Yeah that would be huge.*

*“A lot of us vets our memories aren’t that great and to receive something on a cell phone like a text message letting me know two days from now I have an appointment, that way I wouldn’t forget about it. Yeah, that would be very helpful.”*

Perhaps because of the familiarity with this mode (some used to receive phone call reminders on landlines), there was considerable openness to greater use of mobile phone call reminders. As mentioned above, several preferred live phone calls which were viewed as more personal and allowed greater interactivity, even if they cost more than a text message or an automated call, *“It’s just more personal and you can ask questions.”* Automated calls, while acceptable to some, were viewed more ambiguously by many others. Some referred to them disparagingly as “robo-calls.”

*When you answer it you can’t shut it off and you’re stuck with it and it eats up all your time and you don’t know who it is.*

Also, others noted automated calls can be confusing because they often do not indicate who is calling, which doctor’s office it is coming from, or which clinic to go to, and they often do not provide a phone number to call if one has questions.

Mobile phone text message reminders were seen as practical and efficient. Respondents liked the fact that they provide a written record that one can review as many times as needed. If English is not the recipient’s first language, noted one respondent, the recipient can show the text message to a friend for help in understanding the message content.

*Well you have something solid in front of you. You don’t have to write it down. You can save it and it’s there. I mean you have all your information right there.*

Conversely, a provider or receptionist may have an accent that is hard to understand, as noted one respondent, and a text message would be preferable to voice communication that the patient has a hard time understanding.

Participants liked the asynchronous nature of texting. One reported that he was not supposed to receive phone calls during work, but with text messages he could read and respond to them during breaks or after work. Other respondents suggested that text message reminders should have the option for the recipient to respond, for example to confirm they will attend the appointment, or to request rescheduling or cancellation.

Some in our sample were not sure of the value of text message appointment reminders. Some did not want to receive any reminders (phone or text) on their mobile phone because of the cost. Others were concerned about the potential annoyance of receiving too many text reminders, while others reported they did not view their text in-box frequently enough for it to be valuable as a reminder system for appointments coming in the next one to two days. Some respondents, who lacked skills or confidence in texting, felt they would need to learn to use it because it was a technology that, as one respondent said, is “here to stay.”

#### Medication related reminders

Participants were asked about two kinds of medication-related reminders—for prescription refills and for medication adherence. Medication refill reminders were generally positively seen, whether it was a live person making the reminder call, an automated call, or a text message. Several participants expressed interest in reminders to assist with medication adherence (Table 3).

Participants were generally in favor of receiving mobile phone calls—live or automated—to remind patients that it was time to refill a prescription. Here a respondent describes his preference for live calls for appointment reminders,

*“It’s just more personal and you can ask questions. It’s more informative, just better to talk to a live person*.”

There was also widespread support for text message reminders for medication refills. Respondents proposed other ideas as well. One suggestion concerned hospital pharmacies. When patients were on-site at the hospital or medical center waiting for their medication, the pharmacy could text the patient that their prescription was ready to be picked up at the pharmacy window. This would allow patients to leave the pharmacy waiting room to go to other parts of the medical center, e.g., to cafeteria or store. Several respondents were supportive of text messages used for medication taking, for example the sending of text messages each morning at a specific time to remind a patient to take their pills.

Some respondents expressed concern about potentially receiving too many medication-related reminders. Some said it would be annoying to receive reminders daily or more often, for example before each dose of medication.

*If they do (text messages) more than once I would get annoyed. You know, …if they do it like four, five times …I’ll probably throw the phone up against the wall!*

#### Laboratory results

Participants felt it would be valuable to be notified via their mobile phone that laboratory results were ready to be discussed with a healthcare provider, though this support was not as strong as for appointment reminders (Table 3). While several liked this idea, some were not interested because the current system of receiving lab results from their clinician during office visits worked fine for them, while others felt a text message saying their lab results were ready would just make them feel nervous that the results were going to be bad. Similar to appointment reminders, there was support, however, for text message reminders to have lab work done:

*(Text messages to remind you to have lab work done) would be very helpful because they usually schedule (lab work) like months in advance. And if you don’t write it down on the calendar and you try to remember something like that, it’s impossible. So that would be a very good idea.*

#### Caring outreach

We explored the idea of a check-in or caring outreach, by phone call (live or automated) or text message. One type of message would target patients who had not been seen by their healthcare team in a long time, for example one or two years. The content of such a phone or text outreach was described as, “We were wondering how you are doing because we haven’t seen you in a while? It would be great to see you. Please call xxx-xxx-xxxx to set up an appointment.” Respondents supported this idea (Table 3).

Interviewers also asked about daily outreach efforts delivered via mobile phone, such as “Are you doing alright today?” This was also seen positively. Respondents indicated this would be especially valuable for people who were having psychological difficulties.

*That would be a huge help and then if you say, “no,” (I’m not doing well) well okay then they transfer you and then either you’re texting or phoning with somebody to try to help get you immediate help. Yeah that would be huge.*

## Discussion

Through qualitative interviews with 30 homeless veterans living in a large Northeastern US metropolitan area, we found that the majority had access to, and used, mobile phones and other information technologies regularly, and also reported positive attitudes about health providers using these technologies to connect with homeless veterans related to their healthcare needs. Respondents viewed reminders especially favorably whether for upcoming health appointments, medication refills, medication taking, or to receive laboratory results. There was also support for mobile phone calls or text messages to reach out to individuals whose health was considered at especially high risk.

Life is disrupted when one does not have a stable home and this interrupts health seeking behavior (Gelberg et al., 1997; O’Toole et al., 2007). While some homeless shelters have clinics on site, or facilitate transportation to health care services, in general the US health care system, including the VA health care system, is designed for people who have stable housing, a dependable mailing address, a landline phone, and easy access to the Internet. Homeless veterans lack many of these resources. However, despite the economic, societal, situational and psychological barriers that affect homeless veterans accessing healthcare, our results suggest that IT such as mobile phones can contribute to improving access to outpatient health care services, which in turn may lead to improved health outcomes. While these technologies may not directly address these serious underlying challenges, IT does make it easier to connect with health care providers (e.g., text messages, mobile phone calls, emails and secure messages), remember appointment times (e.g., using calendar and reminder functions on one’s phone, or receiving text message reminders from health care team), and know when it is time to refill a medication (interactive voice response, text messages or email refill reminders).

Treatment engagement is an area that can be addressed through mobile technologies as missed visits and being lost to follow up present a significant problem for managing chronic conditions, including homeless persons trying to manage high-risk health care needs such as HIV, substance abuse, chronic pain, and depression (Giordano et al., 2005; Schluger et al., 1995; Karter et al., 2004; Macharia et al., 1992; Haynes, McDonald & Garg, 2002; Mugavero et al., 2012; Hwang et al., 2011; Weiser et al., 2006). Fortunately, there is evidence that even modest interventions can help improve visit attendance for vulnerable populations, including brochures and posters in exam and waiting rooms reminding patients about the importance of coming to all clinic visits (Gardner et al., 2012), and text message appointment reminders which have been shown to increase appointment attendance in a variety of health care settings with diverse populations (Guy et al., 2012; McInnes et al., 2014). Our finding that many homeless persons use mobile phones suggests this may be a worthwhile approach to reducing missed visits in that population. While text messaging to patients is not currently approved in VA health care, there is a VA texting system in development (McInnes et al., 2014). Data such as presented in this study can encourage the more rapid spread of the system and provide some insights into how it might be used with certain veteran populations.

Another area ripe for innovations is medication adherence. Poor medication adherence is a serious barrier to chronic disease management (Viswanathan et al., 2012), and low income populations are at especially high risk for non-adherence (Mojtabai & Olfson, 2003). Studies of homeless populations’ medication adherence indicate that both patients and providers recognize it is a major problem (Hwang et al., 2011). In a study of homeless and unstably housed tuberculosis patients, 36% reported they expected to have difficulty regularly taking their tuberculosis medications, and 30% said they had no one to help remind them to take medications (Craig et al., 2007). This illustrates the lack of social support networks available to many homeless and unstably housed persons, and the potential role that IT, such as texting, emails, and social media, can play in creating an electronically enhanced social network or system that can support disease self-management. A number of studies in non-homeless populations, including randomized trials, have indicated that text message medication adherence reminders contribute to improved anti-retroviral medication adherence and reductions in viral load for persons with HIV (Lester et al., 2010). Other studies have shown that text messaging interventions can contribute to other behavior changes, such as smoking cessation (Free et al., 2013; Free et al., 2011; Free et al., 2009; Rodgers et al., 2005), blood glucose monitoring by diabetics (Hanauer et al., 2009), and weight loss behaviors (Patrick et al., 2009). While our respondents reacted favorably to the idea of mobile phone medication-related reminders, they warned against over-utilization of such reminders. Once-a-day text-messages seemed to be the maximum acceptable number for medication adherence reminders and other health-related purposes.

It is important to underscore that the use of IT with vulnerable populations is not without challenges. With a low-income population such as the homeless, cost will inevitably be an issue—some of our study participants felt that things like appointment reminders would be a waste of their monthly allotment of talk and text. Other barriers for this population include the difficulty keeping phones charged (Le Dantec & Edwards, 2008), loss and theft of phones (Bure, 2006), and, for those in some emergency shelters, not having access to mobile phones in the evenings because shelters lock up the phones at night. Mental health and substance use disorders, highly prevalent among homeless populations, may also contribute to barriers to health IT use. For example, MH and SUD conditions may interfere with remembering to purchase more minutes or to recharge a phone battery. They may also interfere with income earning and thus lead to diminished financial resources to buy computers and other devices, or to pay monthly internet and phone plan fees. A broader health system issue regarding mobile phone texting relates to privacy. Some health care settings, including the Department of Veterans Affairs, have not approved mobile phone texting because it is considered unsecure (Hassinen & Laitinen, 2005). These systems hope to avoid inadvertent disclosure of sensitive information, for example a patient sending a text message to a provider asking when HIV test results would be ready would be exposing him/herself to loss of privacy (Lim et al., 2008).

There are several limitations to this study. As a small study, using a convenience sample of homeless veterans from a single metropolitan area, the findings may not be generalizable to other populations of homeless veterans in other parts of the country, in rural areas, or to non-veterans. That said, there are many similarities between veteran and non-veterans who are homeless in terms of their health, health care needs, and utilization of services, though some research indicates morbidity may be greater in homeless veterans than in homeless non-veterans (O’Toole et al., 2003). Additionally, we did not interview any homeless veterans living on the streets, living doubled-up with friends or relatives, or using single-room occupancy hotels. Based on results from studies of non-veterans (Eyrich-Garg, 2010; Eyrich-Garg, 2011), we suspect we would find that veterans living on the streets are less likely to own information technologies, and for those that do they likely experience more barriers to regular use of those technologies (e.g., device not charged, lack of internet access, lacking phone minutes). In addition, most participants were males, so the findings may not apply to women. All data collected was via self-report, so the various uses of IT were not verified in an objective manner. Social desirability bias may have caused respondents to speak more favorably of proposed uses of technologies than they actually felt.

### Conclusion

Many homeless veterans have mobile phones and regularly access the internet. Mobile phones may represent an effective tool for communicating with homeless persons and increasing their engagement in care and adherence to treatment. When asked about a variety of kinds of communication, there was support for mobile phone calls and text messages, for purposes such as appointment reminders, medication refill reminders, and reminders to take medications. There was also support for outreach to bring back into care homeless patients who had not been seen by their providers in a long time. Additional research is needed to evaluate implementation of mobile phone communication systems for homeless persons in clinical settings and to assess their impact on engaging homeless persons in health care services.

Mobile technologies may have certain advantages for this population, but PHRs also have the potential to be useful. Further research should examine, for example, the effectiveness of peer-navigators who would help homeless persons access PHRs by providing mini-trainings, technical support, and encouragement to try additional features. This could happen at medical centers—especially ones that have clinics that target homeless patients, or in other places that internet-connected computers are available such as in some shelters, transitional housing, and libraries.

It is important to recall that among persons who are homeless there is a substantial burden of chronic physical and mental health conditions; many in this population would benefit from more frequent assistance from the health care system. Their current situation of housing instability, however, interferes with access to and utilization of needed health services. Innovative uses of technologies are needed that are acceptable to persons who are homeless, feasible given the limited resources they have, and potent enough to change health-related behaviors, processes, and outcomes. Technologies that can increase patient chronic disease self-management and encourage regular follow-up outpatient visits, or even deliver (or augment) mental health therapy are needed. Technologies, for example, can support better adherence to anti-psychotic medications, and be tailored to deliver automated (voicemail or text message) (Burns et al., 2011) or live therapeutic support based on the individual needs and preferences of patients.

## Supplemental Information

10.7717/peerj.1096/supp-1 Supplemental Information 1 The informed consent form used in this study

10.7717/peerj.1096/supp-2 Supplemental Information 2 Approval letter for the study, from the institutional review board

## Additional Information and Declarations

### Data Availability

The following information was supplied regarding the deposition of related data: The informed consent form signed by research subjects does not permit the public sharing of their raw data.
