# Supplementary material for: Preliminary needs assessment of mobile technology use for healthcare among homeless veterans
Source: PeerJ. 2015 Jul 30;3:e1096. doi: 10.7717/peerj.1096 (PMC4525686; doi:10.7717/peerj.1096)
Supplement: Supplemental Information 1 [file peerj-03-1096-s001.pdf]

Participant Name: ______________________ **Date:** ______________

Social Security Number: ______________________ **VAMC:** Bedford, MA

Principal Investigator: D. Keith McInnes, ScD

Title of Study: Information Technology Access and Use Among Homeless Veterans

Sponsor of the Study: National Center on Homelessness among Veterans (NCHV), Bedford Site

1. Purpose of study and how long it will last: You are invited to be in a research study designed to learn about cell phone and other technology use by veterans who have experienced homelessness. We will do one interview with you and this will last about one hour.

2. Description of the study including procedures to be used: In this study you will be interviewed by a researcher who will ask you questions about your use of cell phones, the internet, and other forms of technology. You will also be asked whether you would be interested in using cell phone and internet to keep in touch with VA health care providers, and to get information about your health from the VA, such as seeing a list of medications you are taking or seeing a list of upcoming appointments. There are also questions about you, personally, such as your age, race, income, and where you've lived recently. There will be a total of up to 90 veterans taking part in this study.

If you decide to participate in this research study, the following will happen:

You will be interviewed once, privately, by a research team member. He/she will ask you questions and he/she will write down your answers. In addition, with your permission the researcher will audio record the interview with you to help us remember what you said. There is a separate form we will ask you to sign that gives us permission to record the interview. The interview will last about one hour.

3. Description of any procedures that may result in discomfort or inconvenience: You may feel tired or bored answering the questions. Also some questions may make you feel uncomfortable; you can refuse to answer questions or can choose to withdraw from the study at any time.

4. Expected risks of study: Participation in research may involve a loss of privacy. Your research records will be kept as confidential as possible. Only a code number will identify your research records. The code number will not be based on any information that could be used to identify you (for example, social security number, initials, birth date, etc.) The master list linking names to code numbers will be kept separately from the research data.

All research information will be kept in locked files at all times. Your identity will not be revealed in any reports or publications resulting from this study. Only authorized persons will have access to the information gathered in this study. Federal Agencies such as the Office for Human Research Protection (OHRP), Government Accountability Office (GAO) and Food and

IRB APPROVAL
12/8/11
EXPIRES
10/31/12 DJ

Participant Name: ______________________________ **Date:** _______________

Social Security Number:______________________ **VAMC:** Bedford, MA

Principal Investigator: D. Keith McInnes, ScD

Title of Study: Information Technology Access and Use Among Homeless Veterans

Sponsor of the Study: National Center on Homelessness among Veterans (NCHV), Bedford Site

Drug Administration (FDA) may have access to the records. The Department of Veterans Affairs (VA) requires some information to be recorded in the VA electronic medical record for all veteran and non-veteran research subjects. Therefore, if you participate in this study, a medical record will be created if you do not already have one. Notes from your visits, procedures, and laboratory tests will be included in this record. In addition to the research team, and the VA staff who provide clinical services, other researchers may be granted approval to access this information in the future. Federal laws and regulation that protect privacy of medical records will apply to your VA record.

If you participate this will not in any way affect your regular VA health care and it will not affect in any way what you pay for VA health care (if you make payments).

If the interviewer finds that the interview questions are hard for you to understand or that they are emotionally very uncomfortable for you, he/she may decide to terminate the interview.

5. Expected benefits of study: There are no direct benefits to you from participating in this research.

6. Use of research results/Confidentiality: The completed questionnaire and written notes from your interview questionnaires, as well as the audio recording and a transcript of the audio recording, will be accessible only to the research team members. The paper questionnaire and paper notes will be stored in a locked cabinet in the office of Keith McInnes, ScD, the study principal investigator. His office is on the campus of the Bedford VA Medical Center. Audio recordings and transcripts will be placed on a Bedford VA medical center computer that is password protected. The questionnaire answers will be typed into the same Bedford VA medical center computer as the rest of the study information, and protected by password. Besides Dr. McInnes, the only other persons with access to this study information are the research team members who are working on this study with him. All data will be retained in accordance with the VA record control schedule, after which they will be destroyed.

If results of this study are reported in medical journals or at meetings, you will not be identified by name, by recognizable photograph, or by any other means without your specific consent. Your medical records will be maintained according to VA requirements.

With your permission, we will audio record our interview with you. Actual audio recordings of your voice will not be used in presentations or otherwise presented in a public forum.

IRB APPROVAL 12/8/11 EXPIRES 10/31/12 DJ

Participant Name: ______________________________ **Date:** _______________

Social Security Number:______________________ **VAMC:** Bedford, MA

Principal Investigator: D. Keith McInnes, ScD

Title of Study: Information Technology Access and Use Among Homeless Veterans

Sponsor of the Study: National Center on Homelessness among Veterans (NCHV), Bedford Site

Transcripts of what you have said will be used for research presentations, but only if the investigators do not feel that the content of what you have said is likely to allow others to identify you.

The audio recording and transcript of our interview with you may be used in other studies after this study is completed. The audio-recording and transcript of your interview will be stored on Bedford VA computers and will be the responsibility of Dr. Keith McInnes. He will ensure that in any other studies when data are presented from your audio-recording or transcript that there is no way for others to be able to identify you.

This protection is not absolute. There are five circumstances in which information identifying you might be released:
1) The researchers will report certain communicable diseases as required by State regulations. These types of reports will be made without your knowledge.
2) The researchers will report current cases of physical or sexual abuse (including child abuse and elder abuse).
3) The researchers may disclose medical information without your permission in the event of an emergency. If you indicate you are in imminent danger of hurting yourself or others, the researchers may need to reveal this in order to protect you or someone else.
4) The researcher may be required by VA regulations or by federal law to disclose information from research records.
5) The researchers are required to allow access to research records when requested by regulatory agencies such as the Food and Drug Administration (FDA) for the purpose s of program evaluation or audit.

7. Special Circumstances: You will be paid $25 in VA canteen vouchers immediately after completing the interview.

8. Additional Elements of Informed Consent: We may want to contact you in the future if we have follow-up questions about this study or to see if you would participate in another closely related study. We would contact you using the phone number in your Bedford VA medical record. If you agree to be contacted in the future please initial the statement below.

I agree to be contacted for future research studies. ________
Participant Initials

IRB APPROVAL
12/8/11
EXPIRES
10/31/12 DJ

Participant Name: ______________________ **Date:** ____________

Social Security Number: ____________________ **VAMC:** Bedford, MA

Principal Investigator: D. Keith McInnes, ScD

Title of Study: Information Technology Access and Use Among Homeless Veterans

Sponsor of the Study: National Center on Homelessness among Veterans (NCHV), Bedford Site

RESEARCH PARTICIPANTS' RIGHTS: I have read, or have had read to me all of the above. ____________________ has explained the study to me and answered all of my questions. I have been told of the risks or discomfort and possible benefits of the study. I have been told of other choices of treatment available to me.

I understand that I do not have to take part in this study, and my refusal to participate will involve no penalty or loss of rights to which I am entitled. I may withdraw from this study at any time without penalty or loss of VA or other benefits to which I am entitled.

The results of this study may be published, but my records will not be revealed unless required by law.

A veteran-participant does not have to pay for care received as a participant in a VA research project except in accordance with federal law. Certain veterans have to pay co-payments for medical care and services provided by VA. In case there are medical problems or questions, I have been told I can call: **Dr. Keith McInnes at 781-687-3507 during the day, and 781-687-2000 after hours. VA Medical Facilities shall provide, or arrange for, necessary medical treatment to a research subject injured as a result of participation in a research project. This does not apply to treatment for injuries due to non-compliance by the subject with the study procedures. If I have any questions, concerns, or complaints regarding my rights as a research subject I may call Joseph Squicciarini at 781-687-2926. No money has been set aside for compensation in case of injury as a result of participating in this study; however, I understand that I would still have the right to file any legal action.**

I understand my rights as a research subject, and I voluntarily consent to participate in this study. I understand what the study is about and how and why it is being done. I will receive a signed copy of this consent form.

____________________ ____________

Participant's Signature **Date**

____________________ ____________________ ____________

Signature of Participant's Representative* **Participant's Representative (print)** **Date**

***Only required if subject not competent.**

____________________ ____________________ ____________

Signature of Investigator **Signature of the person obtaining consent** **Date**

IRB APPROVAL 12/8/11 EXPIRES 10/31/12 DJ
