# Supplementary material for: Preliminary needs assessment of mobile technology use for healthcare among homeless veterans
Source: PeerJ. 2015 Jul 30;3:e1096. doi: 10.7717/peerj.1096 (PMC4525686; doi:10.7717/peerj.1096)
Supplement: Supplemental Information 2 [file peerj-03-1096-s002.pdf]

VA DEPARTMENT OF AFFAIRS

REPORT OF SUBCOMMITTEE ON HUMAN STUDIES

Project/Program Title: New Project: Information Technology Access and Use Among Homeless Veterans

Principal Investigator: D. K. McInnes, ScD

VAMC: Bedford Review Date: October 13, 2011

COMMITTEE FINDINGS:

All of the following criteria must be satisfied in order to approve research under 45 CFR 46.111 or 21 CFR 56.111:

1. The information given in the Informed Consent under the Description of Research by Investigator is complete, accurate, and understandable to a research subject or a surrogate who possesses standard reading and comprehension skills.	☒ YES<br>☐ NO<br>☐ N/A
2. Legally effective informed consent will be obtained by the principal investigator or a trained and supervised designate under suitable circumstances that minimize the possibility of coercion or undue influence and give the prospective participant or representative sufficient opportunity to consider whether or not to participate.	☒ YES<br>☐ NO<br>☐ N/A
3. Informed consent will be appropriately documented.	☒ YES<br>☐ NO<br>☐ N/A
4. The risks to participants are minimized by using procedures which are consistent with sound research design and which do not unnecessarily expose participants to risk.	☒ YES<br>☐ NO
5. The risks to participants are minimized whenever appropriate, by using procedures already being performed on the participants for diagnostic or treatment purposes.	☒ YES<br>☐ NO
6. The risks to participants are reasonable in relation to anticipated benefits, if any, to participants, and the importance of the knowledge that may reasonably be expected to result.	☒ YES<br>☐ NO
7. The selection of participants is equitable taking into account the purposes of the research, the setting in which the research will be conducted, the special problems of research involving vulnerable populations, the selection criteria, and the recruitment procedures.	☒ YES<br>☐ NO
8. If subject is incompetent and surrogate consent is obtained, all following conditions have been met; a) the research can't be done on competent subjects; b) there is no risk to the subject, or if risk exists the direct benefit to subject is substantially greater; c) if an incompetent subject resists, he will not have to participate; d) if there exists any question about the subject's competency, the basis for decision on competency has been fully described.	☐ YES<br>☐ NO<br>☒ N/A
9. When some or all of the participants are likely to be vulnerable to coercion or undue influence, such as children, prisoners, pregnant women, mentally disabled persons, or economically or educationally disadvantaged persons, are additional safeguards included in the study to protect the rights and welfare of these participants? (*Note: Subpart B of the DHHS regulations specifies additional protections for pregnant women; Subpart C of the DHHS regulations, for prisoners; and Subpart D of the DHHS and FDA regulations, for children.)*	☒ YES<br>☐ NO<br>☐ N/A

Project/Program Title: New Project: Information Technology Access and Use Among Homeless Veterans

Principal Investigator: D. K. McInnes, ScD

VAMC: Bedford Review Date: October 13, 2011

10. If the subject is paid, the payment is reasonable and commensurate with the subject's contribution?

☒ YES
☐ NO
☐ N/A

11. When appropriate, the research plan makes adequate provision for monitoring the data collected to ensure the safety of participants.

☒ YES
☐ NO
☐ N/A

12. When appropriate, there are adequate provisions to protect the privacy of participants.

☒ YES
☐ NO
☐ N/A

13. When appropriate, there are adequate provisions to maintain the confidentiality of data.

☒ YES
☐ NO
☐ N/A

14. If information has arisen that might affect the willingness of participants to continue to take part in the research, will it be provided to those participants?

☒ YES
☐ NO
☐ N/A

Comments: (Indicate if Expedited Review)
Data repository to be established if data is to be re-used; unused data to be kept according to VA regulations

☐ Expedited ☐ HIPAA Waiver ☐ Informed Consent Waiver

RECOMMENDATION: ☒ APPROVE ☐ DISAPPROVE/REVISE

SIGNATURE OF CHAIRPERSON or CO-CHAIRPERSON	DATE
John M. Wells, PhD/Paula Mroz, PhD [signature]	11/10/11<br>Expire 10/31/12
